# Pathway Analysis for Genome-Wide Association Study of Lung Cancer in Han Chinese Population

**DOI:** 10.1371/journal.pone.0057763

**Published:** 2013-03-01

**Authors:** Ruyang Zhang, Yang Zhao, Minjie Chu, Chen Wu, Guangfu Jin, Juncheng Dai, Cheng Wang, Lingmin Hu, Jianwei Gou, Chen Qian, Jianling Bai, Tangchun Wu, Zhibin Hu, Dongxin Lin, Hongbing Shen, Feng Chen

**Affiliations:** 1 Department of Epidemiology and Biostatistics and Ministry of Education (MOE) Key Lab for Modern Toxicology, School of Public Health, Nanjing Medical University, Nanjing, China; 2 State Key Laboratory of Molecular Oncology and Department of Etiology and Carcinogenesis, Cancer Institute and Hospital, Chinese Academy of Medical Sciences and Peking Union Medical College, Beijing, China; 3 Section of Clinical Epidemiology, Jiangsu Key Laboratory of Cancer Biomarkers, Prevention and Treatment, Cancer Center, Nanjing Medical University, Nanjing, China; 4 Institute of Occupational Medicine and Ministry of Education, Key Laboratory for Environment and Health, School of Public Health, Tongji Medical College, Huazhong University of Science and Technology, Wuhan, China; 5 State Key Laboratory of Reproductive Medicine, Nanjing Medical University, Nanjing, China; Cincinnati Children’s Hospital Medical Center, United States of America

## Abstract

Genome-wide association studies (GWAS) have identified a number of genetic variants associated with lung cancer risk. However, these loci explain only a small fraction of lung cancer hereditability and other variants with weak effect may be lost in the GWAS approach due to the stringent significance level after multiple comparison correction. In this study, in order to identify important pathways involving the lung carcinogenesis, we performed a two-stage pathway analysis in GWAS of lung cancer in Han Chinese using gene set enrichment analysis (GSEA) method. Predefined pathways by BioCarta and KEGG databases were systematically evaluated on Nanjing study (Discovery stage: 1,473 cases and 1,962 controls) and the suggestive pathways were further to be validated in Beijing study (Replication stage: 858 cases and 1,115 controls). We found that four pathways (achPathway, metPathway, At1rPathway and rac1Pathway) were consistently significant in both studies and the *P* values for combined dataset were 0.012, 0.010, 0.022 and 0.005 respectively. These results were stable after sensitivity analysis based on gene definition and gene overlaps between pathways. These findings may provide new insights into the etiology of lung cancer.

## Introduction

Lung cancer is one of the most frequently diagnosed cancers and the leading causes of cancer death globally [1]. In China, the incidence and mortality rates of lung cancer have been increasing rapidly in the last three decades, primarily because of tobacco consumption [2]. However, genetic factors also play an important role in lung carcinogenesis. Over the past several years, genome-wide association studies (GWAS) have identified more than 10 loci associated with lung cancer risk with a modest effect for each single nucleotide polymorphism (SNP) [3], [4], [5], [6], [7], [8]. However, these variants accounted for only a small fraction of hereditability of lung cancer [9], [10], [11].

Given that gene-gene interactions may contribute to complex diseases, it has been suggested that combining the multiple variants with small effect together based on biological pathways using the GWAS data may tend to detect the joint effects of multiple genes and to highlight the specific pathway aggregated in a certain disease [12]. A large proportion of disease susceptibility genes may be functionally related and/or interact with each other in biological pathways and only a small number of biological pathways may mainly contribute to the etiology of complex disease [13]. Thus, pathway-based approaches have been applied to the GWAS of several complex diseases, and some novel disease-susceptibility pathways have been revealed [14], [15], [16], [17], [18], [19], [20], [21], [22], [23]. Recently, Chung et al. (2012) [24] evaluated pathways associated with lung cancer risk in subjects collected by American Cancer Society across all U.S. states using a two-stage random forest-based pathway analysis method based on KEGG database (URL: http://www.genome.jp/kegg/pathway.html/), and identified 4 pathways associated with lung cancer including p53 signaling pathway. Meanwhile, Fehringer et al. (2012) [25] performed pathway analysis on lung cancer risk in subjects collected from Central Europe, Toronto, Germany and Texas using four different methods based on Gene Ontology (GO) database (URL: http://www.geneontology.org/), and found that the acetylcholine receptor activity pathway was significantly associated with lung cancer risk using two different approaches. However, none of pathway analyses of lung cancer GWAS are reported in populations of non-European ancestry to date.

Several methods have been proposed for pathway analysis [26], and one of the commonly used method is gene set enrichment analysis (GSEA) [16]. Briefly, three steps are used for pathway analysis in GSEA. First, individual-SNP association analysis is conducted to determine the effect for each SNP. Second, the representative SNP with the lowest *P* value is mapped to each gene, and all genes are assigned to predefined biological pathways. Finally, all genes are ranked by their significance, and then are to be evaluated whether a particular group of genes is enriched at the top of the ranked list by chance. As a result, a cluster of biological related SNPs which appeared in the top list may be potentially associated with disease as integration.

In a large-scale GWAS of lung cancer in Han Chinese population, we have already validated suggestive SNPs with a *P* value ≤1.0×10^−4^ in independent populations and found five new lung cancer risk-related loci with effect size (odds ratio) ranging from 0.84 to 1.35 at a genome-wide significance level [3], [4]. To further deeply understand the genetics mechanism of lung cancer and identify the crucial pathway in lung carcinogens, we currently performed a two-stage pathway analysis using GSEA method based on our existing GWAS data in Han Chinese population. In stage 1, we screened all available pathways in Nanjing study using 1,473 cases and 1,962 controls. In stage 2, the pathways with *P* values ≤0.05 and *FDR* ≤0.50 were validated in Beijing study using 858 cases and 1,115 controls.

## Materials and Methods

### Ethics Statement

This collaborative study was approved by the institutional review boards of China Medical University, Tongji Medical College, Fudan University, Nanjing Medical University and Guangzhou Medical College with written informed consent from all participants [27], [28], [29], [30].

### Study Participants

The study subjects were from an ongoing two-center GWAS of lung cancer in China, including Nanjing study and Beijing study. The details of population have been described elsewhere [3]. Briefly, there were 1,473 cases and 1,962 controls in Nanjing study, 858 cases and 1,115 controls in Beijing study after quality control. All lung cancer cases were histopathologically or cytologically confirmed by at least two local pathologists. All controls were selected from those receiving routine physical examinations in local hospitals or those participating in the community screening of noncommunicable diseases and frequency-matched for age, gender and geographic regions to each set of the lung cancer cases.

### Genotyping and Quality Control

Genotyping was performed using an Affymetrix Genome-Wide Human SNP Array 6.0. A systematic quality control procedure was applied for both SNPs and individuals before pathway analysis [3]. Briefly, SNPs were excluded if they did not map on autosomal chromosomes, had a call rate <95%, minor allele frequency (MAF) <0.05, *P*<1×10^−5^ for Hardy-Weinberg equilibrium (HWE) in combined samples of two studies or *P*<1×10^−4^ for HWE in either the Nanjing or Beijing study samples. We removed samples with call rate <95%, ambiguous gender, familial relationships, extreme heterozygosity rate and outliers. Finally, a total of 2,331 cases and 3,077 controls (Nanjing study: 1,473 cases and 1,962 controls; Beijing study: 858 cases and 1,115 controls) with 570,373 SNPs were remained in subsequent pathway analysis.

### Pathway Data Construction

We collected pathways from two public resources: KEGG and BioCarta database (URL: http://www.biocarta.com/). Pathways containing genes from 10 to 200 were included in this study. This gene number range was considered appropriate to reduce the multiple-comparison issue and to avoid testing overly narrow or broad functional gene categories [22]. Pathway overlap was defined as the percentage of shared genes to total ones of two pathways [14].

### Statistical Analysis

Logistic regression model with adjustment for age, gender, pack-year of smoking and the first four principal components derived from EIGENSTRAT 3.0 [31] was used to evaluate the association significance of each SNP using GLM package executed in R software (version 2.14.0; The R Foundation for Statistical Computing). SNPs were assigned to a gene if they located within 50 kb downstream or upstream of the gene. The significance of each gene was derived from the representative SNP. All genes were assigned to pathways. Then the association between lung cancer risk and each pathway was evaluated by GenGen software [16] using the weighted Kolmogorov-Smirnov-like running sum statistic (denoted by enrichment score, *ES*), which reflected the over-representation of a cluster of genes within this pathway at the top of the entire ranked list of genes in the genome. We randomly shuffled the case-control status for 1,000 times, and repeated these above steps to get the permuted pathway association results. Thus, the normalized *ES* after adjusted for different sizes of genes, could be acquired via the permutation procedure. Meanwhile, *P* value of each pathway and the false discovery rate (FDR) to keep the proportion of expected false positive findings were derived. The significance level of pathways analysis was set to be *P*≤0.05 and *FDR* ≤0.5.

## Results

### Genetic Information and Prior Biological Information used in Pathway Analysis

Of the total 570,373 genotyped SNPs, 340,060 SNPs were mapped into 17,225 genes within 50 kb upstream or downstream. Among them, 135,160 SNPs were ultimately assigned to 3,514 genes within the pre-defined pathways (41,560 SNPs to 1,003 genes in BioCarta pathways and 120,864 SNPs to 3,134 genes in KEGG pathways). All genes were assigned to 368 pathways that contained 10 to 200 genes (191 pathways used in BioCarta database, 177 pathways used in KEGG database) in following pathway analysis.

### Results of Pathway Analysis using GSEA Method

As presented in **Table 1**, a total 22 pathways were significant (*P*≤0.05, *FDR* ≤0.5) in Nanjing study. Among them, five pathways were successfully replicated in Beijing study, including achPathway (*P* = 0.007), agrPathway (*P* = 0.033), At1rPathway (*P* = 0.003), metPathway (*P* = 0.007) and rac1Pathway (*P* = 0.041). After combining the two studies, four of five replicated pathways remained significance, including achPathway (*P* = 0.012), At1rPathway (*P* = 0.022), metPathway (*P* = 0.010) and rac1Pathway (*P* = 0.005). While, in KEGG database, no pathway was identified (data not shown). Among the significant pathways (*P*≤0.05) for the combined dataset of the two studies (**Table S1 in File S1**), these four indentified pathways were ranked in the top list (No.1 for rac1Pathway, No.3 for metPathway, No.4 for achPathway and No.5 for At1rPathway).

**Table 1:** Summary of significant pathways in Nanjing study (*P*≤0.05 and *FDR*≤0.5) and replication in Beijing study.

Pathway	Description	Gene Count	Nanjing Study			Beijing Study		Combined	
			*NES*	*P*	*FDR*	*NES*	*P*	*NES*	*P*
g1Pathway	Cell Cycle: G1/S Check Point	27	3.29	0.001	0.10	−0.29	0.589	0.88	0.195
**achPathway**	Role of nicotinic acetylcholine receptors in the regulation of apoptosis	16	2.96	0.002	0.11	2.72	**0.007**	2.22	**0.012**
ctcfPathway	CTCF: First Multivalent Nuclear Factor	23	3.07	0.002	0.11	−0.17	0.568	0.96	0.173
agrPathway	Agrin in Postsynaptic Differentiation	31	2.61	0.004	0.23	1.94	**0.033**	0.77	0.204
ecmPathway	Erk and PI-3 Kinase Are Necessary for Collagen Binding in Corneal Epithelia	23	2.36	0.010	0.31	1.56	0.066	1.16	0.128
erythPathway	Erythrocyte Differentiation Pathway	15	2.40	0.011	0.33	0.02	0.474	1.87	0.029
rhoPathway	Rho cell motility signaling pathway	29	2.24	0.014	0.37	−1.36	0.917	1.36	0.095
**At1rPathway**	Angiotensin II mediated activation of JNK Pathway via Pyk2 dependentsignaling	28	2.12	0.017	0.39	2.72	**0.003**	1.97	**0.022**
arfPathway	Tumor Suppressor Arf Inhibits Ribosomal Biogenesis	16	2.08	0.017	0.39	0.53	0.311	0.40	0.335
**metPathway**	Signaling of Hepatocyte Growth Factor Receptor	32	2.16	0.019	0.40	2.20	**0.007**	2.41	**0.010**
srcRPTPPathway	Activation of Src by Protein-tyrosine phosphatase alpha	11	1.89	0.025	0.43	−0.32	0.614	1.77	0.030
hcmvPathway	Human Cytomegalovirus and Map Kinase Pathways	17	1.91	0.030	0.45	0.55	0.301	0.47	0.311
telPathway	Telomeres, Telomerase, Cellular Aging, and Immortality	18	1.86	0.031	0.43	−1.61	0.946	0.22	0.411
cellcyclePathway	Cyclins and Cell Cycle Regulation	23	2.02	0.032	0.41	−0.44	0.654	0.81	0.208
cxcr4Pathway	CXCR4 Signaling Pathway	24	1.79	0.033	0.42	1.26	0.100	0.80	0.205
p38mapkPathway	p38 MAPK Signaling Pathway	31	1.83	0.037	0.44	1.61	0.057	−0.35	0.618
il17Pathway	IL 17 Signaling Pathway	15	1.91	0.037	0.48	−0.78	0.786	0.25	0.396
**rac1Pathway**	Rac 1 cell motility signaling pathway	23	1.82	0.040	0.42	1.85	**0.041**	2.63	**0.005**
edg1Pathway	Phospholipids as signalling intermediaries	26	1.72	0.043	0.44	1.60	0.054	0.50	0.305
skp2e2fPathway	E2F1 Destruction Pathway	10	1.73	0.046	0.46	1.24	0.113	2.58	0.006
ptc1Pathway	Sonic Hedgehog (SHH) Receptor Ptc1 Regulates cell cycle	11	1.72	0.047	0.42	0.20	0.416	1.61	0.062
gleevecpathway	Inhibition of Cellular Proliferation by Gleevec	23	1.66	0.050	0.43	−1.03	0.841	−0.27	0.605

For the achPathway, 9 of 16 ones had SNPs with *P* values <0.05; similarly, 15, 14 and 18 significant genes were observed among 28 genes in the At1rPathway, 32 genes in the metPathway and 23 genes in the rac1Pathway, respectively. The representative SNPs with lung cancer risk at *P* values <0.01 for each gene in these 4 pathways shown in **Table 2**.

**Table 2:** Association results of representative SNPs at *P*<0.01 for genes in four identified pathways.

Pathway	Gene	SNP	CHR	Position	*OR* (95%*CI*)^a^	*P* a
achPathway	PTK2	rs12544802	8	142012928	1.45 (1.23, 1.72)	1.26E−05
	TERT	rs2736100	5	1339516	1.26(1.14, 1.39)	3.73E−06
	RAPSN	rs3781626	11	47399469	0.86 (0.78, 0.94)	6.20E−04
	PIK3R1	rs4122269	5	67581380	0.84 (0.75, 0.93)	7.05E−04
	PTK2B	rs17057065	8	27298991	0.85 (0.77, 0.95)	2.75E−03
	SRC	rs17194885	20	35501803	1.14 (1.04, 1.25)	5.13E−03
	BAD	rs11231735	11	63796574	0.89 (0.82, 0.97)	7.93E−03
At1rPathway	PTK2	rs12544802	8	142012928	1.45 (1.23, 1.72)	1.26E−05
	EGFR	rs11976696	7	55199827	1.17 (1.07, 1.27)	4.90E−04
	PRKCA	rs9896905	17	62206603	0.72 (0.60, 0.88)	8.67E−04
	PRKCB	rs9940072	16	23795684	1.14 (1.06, 1.24)	1.63E−03
	MAP3K1	rs16886403	5	56175003	0.87 (0.80, 0.95)	1.90E−03
	PAK1	rs1237490	11	76722904	0.87 (0.79, 0.95)	2.46E−03
	RAC1	rs2689420	7	6376846	1.23 (1.07, 1.40)	2.47E−03
	PTK2B	rs17057065	8	27298991	0.85 (0.77, 0.95)	2.75E−03
	GNAQ	rs10512065	9	79815464	1.16 (1.05, 1.29)	3.71E−03
	SRC	rs17194885	20	35501803	1.14 (1.04, 1.25)	5.13E−03
	MEF2D	rs12136856	1	154739738	1.13 (1.03, 1.23)	7.70E−03
metPathway	PTK2	rs12544802	8	142012928	1.45 (1.23, 1.72)	1.26E−05
	CRKL	rs178260	22	19654308	1.20 (1.10, 1.30)	2.11E−05
	PTPN11	rs6489847	12	111443096	1.33 (1.16, 1.53)	6.82E−05
	PIK3R1	rs4122269	5	67581380	0.84 (0.75, 0.93)	7.05E−04
	DOCK1	rs11245197	10	128537297	1.16 (1.06, 1.27)	1.29E−03
	RAP1A	rs4838920	1	112046582	0.82 (0.72, 0.93)	1.89E−03
	RAPGEF1	rs11243445	9	133463092	1.25 (1.08, 1.44)	2.43E−03
	PAK1	rs1237490	11	76722904	0.87 (0.79, 0.95)	2.46E−3
	PTK2B	rs17057065	8	27298991	0.85 (0.77, 0.95)	2.75E−03
	ITGA1	rs889295	5	52146467	1.14 (1.04, 1.24)	4.89E−03
	CRK	rs7213811	17	1256997	0.89 (0.81, 0.97)	8.38E−03
rac1Pathway	RALBP1	rs9960523	18	9446982	1.26 (1.12, 1.42)	1.57E−04
	TRIO	rs386450	5	14159136	1.17 (1.08, 1.28)	2.33E−04
	CDK5	rs2069443	7	150386106	1.18 (1.08, 1.29)	3.52E−04
	MYLK	rs2700380	3	125091101	0.85 (0.78, 0.93)	4.78E−04
	PIK3R1	rs4122269	5	67581380	0.84 (0.75, 0.93)	7.05E−04
	WASF1	rs7761436	6	110609524	1.15 (1.06, 1.26)	1.49E−03
	MAP3K1	rs16886403	5	56175003	0.87 (0.80, 0.95)	1.90E−03
	PAK1	rs1237490	11	76722904	0.87 (0.79, 0.95)	2.46E−03
	RAC1	rs2689420	7	6376846	1.23 (1.07, 1.40)	2.47E−03
	CFL1	rs659824	11	65393085	0.85 (0.76, 0.95)	4.48E−03
	ARFIP2	rs2253104	11	6456885	1.17 (1.04, 1.30)	6.25E−03
	LIMK1	rs810549	7	73131469	1.14 (1.03, 1.25)	9.20E−03

a Derived from logistic regression model with adjustment for age, gender, pack-year of smoking and principal components in combined dataset of Nanjing and Beijing studies.

### Sensitivity Analysis of Pathway-based Association

In order to evaluate the influence of SNP-to-gene mapping strategy on pathway analysis, we further mapped SNPs to genes within 20 kb upstream or downstream. Twenty-three pathways were significant in Nanjing study, and 7 of them were replicated in Beijing study. After combining two studies together, three pathways (achPathway, metPathway and rac1Pathway) were still significant (0.019, 0.005 and 0.004 respectively) (**Table S2 in File S1**). These three pathways were also identified in the initial analysis approach. In addition, At1rPathway that was identified in the initial analysis was also significant for combined dataset (*P* = 0.015) though the *P* value was 0.086 in Nanjing study. For the significant pathways observed in Nanjing study, 19 pathways were detected in both approaches using ‘50 kb’ rule and ‘20 kb’ rule and the concordance rate was 73.08% (19/26) between two approaches.

Considering the overlap of genes between pathways, we calculated the pair-wise overlaps between 4 identified pathways and found the overlap rates ranged from 6.25% to 25.00% (**Table S3 in File S1**). Since only significant genes in pathways contributed to the final results, we re-calculated the overlaps between these pathways using their significant genes, and the overlap rates were from 4.17% to 18.75% (**Table S3 in File S1**). Furthermore, four genes (*PAK1*, *PIK3R1*, *PTK2*, and *PTK2B*) that contributed to more than 2 pathways (**Table S4 in File S1**), were further removed from pathways for sensitivity analysis. As a result, the four identified pathways (achPathway, metPathway, At1rPathway and rac1Pathway) were still significant (**Table S5 in File S1**) in combined dataset (*P* = 0.033, 0.040, 0.039 and 0.012, respectively).

## Discussion

GWAS have successfully identified a number of loci associated with diseases/traits, which have greatly improved our understanding on genetic mechanism of these phenotypes. However, as the stringent quality control procedure and strict correction on multiple comparisons are used in GWAS, individual SNPs that are truly associated with phenotypes with modest effect may have been lost. Therefore, a pathway-based approach that evaluates the cumulative contribution of the function-related genes may provide new insights into the biology of a certain disease utilizing GWAS data. GSEA has two major advantages compared with other methods [16], [26]. First, it performs two-step permutation-based correction procedure which effectively adjusts for different sizes of genes and preserves correlations of SNPs in the same gene. Second, covariates such as age, gender or population stratification in GWAS can be adjusted in GSEA. Thus, in the current study, we used GSEA and identified four pathways (achPathway, metPathway, At1rPathway and rac1Pathway) that may play an important role in the development of lung cancer in Han Chinese population. These findings were stable after sensitivity analysis when considering the SNP-to-gene mapping approach and gene overlapping between pathways.

The achPathway (Role of nicotinic acetylcholine receptors in the regulation of apoptosis) was identified to be the top pathways associated with lung cancer risk in this study. Nicotinic acetylcholine receptors (nAchRs) are essential for neuromuscular signaling and have also been found on non-neuronal cells, such as bronchial epithelial cells and lung cancer cell lines [32], [33], [34]. Nicotine and its derived carcinogenic nitrosamines may play an important role in the pathogenesis of lung cancer through the binding to nAChRs expressed in lung epithelial cells, which mainly result from the resistance of cancer cells to apoptosis [35]. Maneckjee et al. (1994) showed that low concentrations of nicotine could block the induction of apoptosis in lung cancer cells [33]. In addition to conferring resistance against apoptosis, several studies have shown that nAChRs can induce cell proliferation as well as angiogenesis [36], [37], both of which are involved in the genesis of cancer.

Importantly, several GWAS based on Caucasian populations have consistently identified 15q25 as lung cancer susceptibility region [5], [6], [7], which contains the nicotinic acetylcholine receptor subunit gene cluster, harboring *CHRNA5*, *CHRNA3* and *CHRNA4* genes. *TERT* is included in the achPathway and its representative SNP rs2736100 has been identified as a lung cancer susceptibility locus in different ethnic populations [3], [8], especially in Asian populations [38], [39], [40].

In the At1rPathway (Angiotensin II mediated activation of JNK Pathway via Pyk2 dependent signaling), Clereet. al (2010) proposed that angiotensin II Type 2 receptor (AT2R) would promote tumor development, including both malignant cell proliferation and tumor angiogenesis [41]. Over-expression of angiotensin II type 2 receptor gene induces cell death in lung adenocarcinoma cells [42], [43]. The aberrant activated JNK pathway can cause pathological cell death and different diseases including cancer [44], while mutations in the JNK pathway can also be involved in cancer development [45].

For the metPathway (Signaling of Hepatocyte Growth Factor Receptor), the hepatocyte growth factor receptor, also called c-Met, is activated by hepatocyte growth factor (HGF). Aberrant c-Met signaling plays significant roles in the pathogenesis and biology of human cancers [46]. Meanwhile, Mutated and over-expressed forms of c-Met are associated with oncogenesis and metastasis, making c-Met a potential therapeutic target for cancer drugs [47], [48]. Interestingly, c-Met and epidermal growth factor receptor (EGFR)inhibitors can synergistically inhibit cell proliferation and promote apoptosis in lung cancer [49]. Yano et al. (2008) proposed that HGF-mediated MET activation can induce gefitinib resistance in lung adenocarcinoma with *EGFR-*activating mutations [50].

For the rac1Pathway (Rac 1 cell motility signaling pathway), Rac-1 is a small GTP-binding protein in the Rho family that regulates cell motility and proliferation in response to extracellular signals [51]. Meanwhile, Rac-1 can function as oncogenes in fibroblasts when over expressed [52]. Rab5 can induce the activation of Rac through several mechanisms, Rab5-regulated trafficking of Rac is involved in cell motility, which may also influence cell migration during morphogenesis and cancer metastasis [53], [54]. Meanwhile, Rac activation by the IRSp53/Eps8 complex plays an important role in the metastatic behavior of the malignant tumor cell [55].

In addition, we also checked the reported pathways by Chung et al. (2012) and Fehringer et al. (2012) in our study. Interestingly, the strongest association reported by Fehringer et al. (2012) was the acetylcholine receptor activity pathway while the achPathway was identified in the current study. Some studies have demonstrated that the activation of nicotinic acetylcholine receptors can alter apoptotic signaling as well as stimulate proliferation, both of which play an important role in lung carcinogenesis [56], [57], [58]. The results consistently support the importance of the 2 pathways in the development of lung cancer, which is biologically plausible. However, we did not found significant signals for other reported pathways. This may be explained by ethnic heterogeneity, different pathway analysis methods or definition of pathways from different databases.

This study has several strengths. First, we performed a two-stage pathway analysis in two independent populations, which may reduce the false-positive findings and improve the credibility of the results. Second, the four identified pathways were still stable after sensitivity analysis when considering SNP-to-gene mapping approaches and gene overlapping between pathways. This point as well as the important biology of the identified pathways in lung carcinogenesis has increased our confidence that our findings may be true other than just by chance. Nevertheless, several limitations are also need to be addressed in this study. First, incomplete annotation of human genome may reduce the study power for the pathway analysis, since many genes of unknown function cannot be assigned to known pathways and intergenic SNPs physically faraway from genes were not included yet. Thus, further studies based on improved genome-annotation database may provide additional understandings in genetics of lung cancer. Second, different pathway databases have different guidelines for pathway construction. Thus, the gene content of pathways representing the same biological process may vary substantially between different databases [14]. We only focused on KEGG and BioCarta databases that may have restricted our analysis due to inherent definition of the pathway, though these two databases have been commonly used in pathway analysis [19], [22], [24]. Finally, it’s better to perform gene-smoking interaction analysis or gene-gene interaction analysis to discuss further on the involvement of these four pathways in smoking-related carcinogenesis. We will investigate them in our future studies.

In summary, this study conducted a two-stage pathway analysis in GWAS of lung cancer in Han Chinese using GSEA method, and identified four pathways (achPathway, metPathway, At1rPathway and rac1Pathway) associated with lung cancer risk. These findings may be an important supplement for GWAS and provide new insights into biology of lung cancer.

## Supporting Information

File S1 **Table S1.** The rank of pathways based on combined dataset of Nanjing and Beijing studies. **Table S2.** Sensitivity analysis of pathway analysis for genes defined by SNPs within 20 kb upstream or downstream. **Table S3.** Gene overlaps between 4 indentified pathways for all genes defined by BioCarta database or genes with significant representative SNPs (*P*<0.05)^a^. (a) The bottom-left of the symmetric matrix is the number of overlap genes between pair-wise pathways and their total gene number. The top-right part is the overlap rate between pair-wise pathways (%). **Table S4.** Genes with significant representative SNPs (*P*≤0.01) contributed to multiple pathways. (a) Derived from logistic regression model with adjustment for age, gender, pack-year of smoking and principal components in combined dataset of Nanjing and Beijing studies. **Table S5.** The results of sensitivity analysis for 4 identified pathway after removing significant overlapping genes (*PAK1*, *PIK3R1*, *PTK2* and *PTK2B*).
(DOCX)
