# Supplementary material for: Pathway Analysis for Genome-Wide Association Study of Lung Cancer in Han Chinese Population
Source: PLoS One. 2013 Mar 1;8(3):e57763. doi: 10.1371/journal.pone.0057763 (PMC3585721; doi:10.1371/journal.pone.0057763)
Supplement: File S1 — Table S1. The rank of pathways based on combined dataset of Nanjing and Beijing studies. Table S2. Sensitivity analysis of pathway analysis for genes defined by SNPs within 20 kb upstream or downstream. Table S3. Gene overlaps between 4 indentified pathways for all genes defined by BioCarta database or genes with significant representative SNPs (P<0.05)a. (a) The bottom-left of the symmetric matrix is the number of overlap genes between pair-wise pathways and their total gene number. The top-right part is the overlap rate between pair-wise pathways (%). Table S4. Genes with significant representative SNPs (P≤0.01) contributed to multiple pathways. (a) Derived from logistic regression model with adjustment for age, gender, pack-year of smoking and principal components in combined dataset of Nanjing and Beijing studies. Table S5. The results of sensitivity analysis for 4 identified pathway after removing significant overlapping genes (PAK1, PIK3R1, PTK2 and PTK2B). (DOCX) [file pone.0057763.s001.docx]

**Supporting Information S1**

**Table S1**. The rank of pathways based on combined dataset of Nanjing and Beijing studies.

| Rank | Pathway | Description | Gene Count | *NES* | *P* | *FDR* |
| --- | --- | --- | --- | --- | --- | --- |
| **1** | **rac1Pathway** | Rac 1 cell motility signaling pathway | 23 | 2.63 | 0.005 | 0.94 |
| 2 | skp2e2fPathway | E2F1 Destruction Pathway | 10 | 2.58 | 0.006 | 0.53 |
| **3** | **metPathway** | Signaling of Hepatocyte Growth Factor Receptor | 32 | 2.41 | 0.010 | 0.55 |
| **4** | **achPathway** | Role of nicotinic acetylcholine receptors in the regulation of apoptosis | 16 | 2.22 | 0.012 | 0.68 |
| **5** | **At1rPathway** | Angiotensin II mediated activation of JNK Pathway via Pyk2 dependent signaling | 28 | 1.97 | 0.022 | 0.82 |
| 6 | pyk2Pathway | Links between Pyk2 and Map Kinases | 27 | 2.08 | 0.024 | 0.76 |
| 7 | arapPathway | ADP-Ribosylation Factor | 16 | 1.85 | 0.029 | 0.71 |
| 8 | erythPathway | Erythrocyte Differentiation Pathway | 15 | 1.87 | 0.029 | 0.78 |
| 9 | srcRPTPPathway | Activation of Src by Protein-tyrosine phosphatase alpha | 11 | 1.77 | 0.030 | 0.77 |
| 10 | p27Pathway | Regulation of p27 Phosphorylation during Cell Cycle Progression | 12 | 1.94 | 0.033 | 0.76 |

**Table S2**. Sensitivity analysis of pathway analysis for genes defined by SNPs within 20 kb upstream or downstream.

| Pathway | Description | Gene Count | Nanjing Study | | | Beijing Study | | Combined | |
| --- | --- | --- | --- | --- | --- | --- | --- | --- | --- |
|  |  |  | *NES* | *P* | *FDR* | *NES* | *P* | *NES* | *P* |
| g1Pathway | Cell Cycle: G1/S Check Point | 27 | 3.64 | 0.001 | 0.02 | -0.08 | 0.518 | 0.61 | 0.255 |
| ctcfPathway | CTCF: First Multivalent Nuclear Factor | 23 | 3.24 | 0.001 | 0.05 | -0.17 | 0.559 | 0.72 | 0.228 |
| **achPathway** | Role of nicotinic acetylcholine receptors in the regulation of apoptosis | 16 | 2.71 | 0.003 | 0.21 | 1.96 | **0.027** | 2.05 | **0.019** |
| agrPathway | Agrin in Postsynaptic Differentiation | 30 | 2.56 | 0.006 | 0.25 | 1.99 | **0.029** | 0.83 | 0.192 |
| ptc1Pathway | Sonic Hedgehog (SHH) Receptor Ptc1 Regulates cell cycle | 11 | 2.32 | 0.007 | 0.20 | -0.11 | 0.542 | 1.42 | 0.076 |
| **metPathway** | Signaling of Hepatocyte Growth Factor Receptor | 31 | 2.33 | 0.008 | 0.22 | 2.20 | **0.010** | 2.86 | **0.005** |
| gleevecpathway | Inhibition of Cellular Proliferation by Gleevec | 22 | 2.39 | 0.008 | 0.23 | -0.46 | 0.675 | 0.85 | 0.204 |
| erythPathway | Erythrocyte Differentiation Pathway | 15 | 2.48 | 0.008 | 0.26 | -0.78 | 0.774 | 1.51 | 0.065 |
| arfPathway | Tumor Suppressor Arf Inhibits Ribosomal Biogenesis | 16 | 2.45 | 0.009 | 0.23 | 0.65 | 0.275 | 1.16 | 0.124 |
| hcmvPathway | Human Cytomegalovirus and Map Kinase Pathways | 16 | 2.37 | 0.009 | 0.22 | 0.23 | 0.423 | 0.57 | 0.295 |
| cxcr4Pathway | CXCR4 Signaling Pathway | 23 | 2.22 | 0.012 | 0.22 | 1.56 | 0.062 | 1.18 | 0.119 |
| rhoPathway | Rho cell motility signaling pathway | 29 | 2.22 | 0.015 | 0.20 | -1.03 | 0.848 | 1.12 | 0.131 |
| il17Pathway | IL 17 Signaling Pathway | 15 | 2.24 | 0.017 | 0.23 | -1.27 | 0.886 | 0.70 | 0.249 |
| ecmPathway | Erk and PI-3 Kinase Are Necessary for Collagen Binding in Corneal Epithelia | 22 | 2.08 | 0.023 | 0.25 | 1.65 | 0.051 | 0.81 | 0.219 |
| tcrPathway | T Cell Receptor Signaling Pathway | 28 | 1.98 | 0.025 | 0.30 | 2.27 | **0.012** | 1.26 | 0.116 |
| **rac1Pathway** | Rac 1 cell motility signaling pathway | 23 | 2.09 | 0.028 | 0.27 | 1.98 | **0.033** | 2.78 | **0.004** |
| telPathway | Telomeres, Telomerase, Cellular Aging, and Immortality | 18 | 1.91 | 0.030 | 0.33 | -1.19 | 0.881 | -0.12 | 0.544 |
| p38mapkPathway | p38 MAPK Signaling Pathway | 31 | 1.91 | 0.031 | 0.32 | 1.14 | 0.130 | -0.23 | 0.584 |
| edg1Pathway | Phospholipids as signalling intermediaries | 25 | 1.76 | 0.033 | 0.38 | 1.30 | 0.095 | 0.10 | 0.466 |
| cellcyclePathway | Cyclins and Cell Cycle Regulation | 23 | 1.88 | 0.037 | 0.32 | 0.28 | 0.387 | 0.93 | 0.186 |
| tffPathway | Trefoil Factors Initiate Mucosal Healing | 19 | 1.82 | 0.038 | 0.35 | 2.51 | **0.005** | 1.47 | 0.073 |
| gsk3Pathway | Inactivation of Gsk3 by AKT causes accumulation of b-catenin in Alveolar Macrophages | 26 | 1.73 | 0.039 | 0.39 | 0.31 | 0.367 | -0.52 | 0.678 |
| vegfPathway | VEGF, Hypoxia, and Angiogenesis | 26 | 1.66 | 0.048 | 0.42 | 1.91 | **0.028** | 1.00 | 0.153 |
| **At1rPathway** | Angiotensin II mediated activation of JNK Pathway via Pyk2 dependent signaling | 27 | 1.41 | 0.086 | 0.43 | 2.78 | **0.003** | 2.10 | **0.015** |

**Table S3**. Gene overlaps between 4 indentified pathways for all genes defined by BioCarta database or genes with significant representative SNPs (*P* < 0.05)^a^.

| **All genes** |  |  |  |  |
| --- | --- | --- | --- | --- |
|  | At1rPathway | metPathway | achPathway | rac1Pathway |
| At1rPathway |  | 25.00 | 7.32 | 6.25 |
| metPathway | 12(48) |  | 11.63 | 7.84 |
| achPathway | 3(41) | 5(43) |  | 8.33 |
| rac1Pathway | 3(48) | 4(51) | 3(36) |  |
|  |  |  |  |  |
| **Significant genes** |  |  |  |  |
|  | At1rPathway | metPathway | achPathway | rac1Pathway |
| At1rPathway |  | 16.67 | 17.65 | 12.50 |
| metPathway | 3(18) |  | 18.75 | 8.33 |
| achPathway | 3(17) | 3(16) |  | 4.17 |
| rac1Pathway | 3(24) | 2(24) | 1(24) |  |

^a^ The bottom-left of the symmetric matrix is the number of overlap genes between pair-wise pathways and their total gene number. The top-right part is the overlap rate between pair-wise pathways (%).

**Table S4**. Genes with significant representative SNPs (*P* ≤ 0.01) contributed to multiple pathways.

| Gene | *P* | Genes contributed to the pathways | | | | Overlaps |
| --- | --- | --- | --- | --- | --- | --- |
|  |  | At1rPathway | metPathway | achPathway | rac1Pathway |  |
| **PAK1** | 2.46E-03 | √ | √ |  | √ | 3 |
| **PIK3R1** | 7.05E-04 |  | √ | √ | √ | 3 |
| **PTK2** | 1.26E-05 | √ | √ | √ |  | 3 |
| **PTK2B** | 2.75E-03 | √ | √ | √ |  | 3 |
| RAC1 | 2.47E-03 | √ |  |  | √ | 2 |
| MAP3K1 | 1.90E-03 | √ |  |  | √ | 2 |
| SRC | 5.13E-03 | √ |  | √ |  | 2 |

^a^ Derived from logistic regression model with adjustment for age, gender, pack-year of smoking and principal components in combined dataset of Nanjing and Beijing studies.

**Table S5**. The results of sensitivity analysis for 4 identified pathway after removing significant overlapping genes (*PAK1*, *PIK3R1*, *PTK2* and *PTK2B*).

| Pathway | Gene Count | Nanjing Study | | | | Beijing Study | | Combined | |
| --- | --- | --- | --- | --- | --- | --- | --- | --- | --- |
|  |  | *NES* | *P* | *FDR* | *FWER* | *NES* | *P* | *NES* | *P* |
| achPathway | 13 | 2.73 | **0.004** | 0.22 | 0.40 | 2.42 | **0.010** | 1.93 | **0.033** |
| metPathway | 28 | 1.64 | **0.045** | 0.55 | 1.00 | 1.53 | 0.059 | 1.76 | **0.040** |
| At1rPathway | 24 | 1.80 | **0.050** | 0.58 | 0.99 | 3.19 | **0.001** | 1.71 | **0.039** |
| rac1Pathway | 20 | 1.52 | 0.069 | 0.57 | 1.00 | 1.37 | 0.090 | 2.30 | **0.012** |
